# Supplementary material for: Microtubule asters anchored by FSD1 control axoneme assembly and ciliogenesis
Source: Nat Commun. 2018 Dec 11;9:5277. doi: 10.1038/s41467-018-07664-2 (PMC6290075; doi:10.1038/s41467-018-07664-2)
Supplement: Supplementary file 2 — Supplementary Information [file 41467_2018_7664_MOESM2_ESM.pdf]

Microtubule asters anchored by FSD1 control axoneme assembly and ciliogenesis

Tu et al.

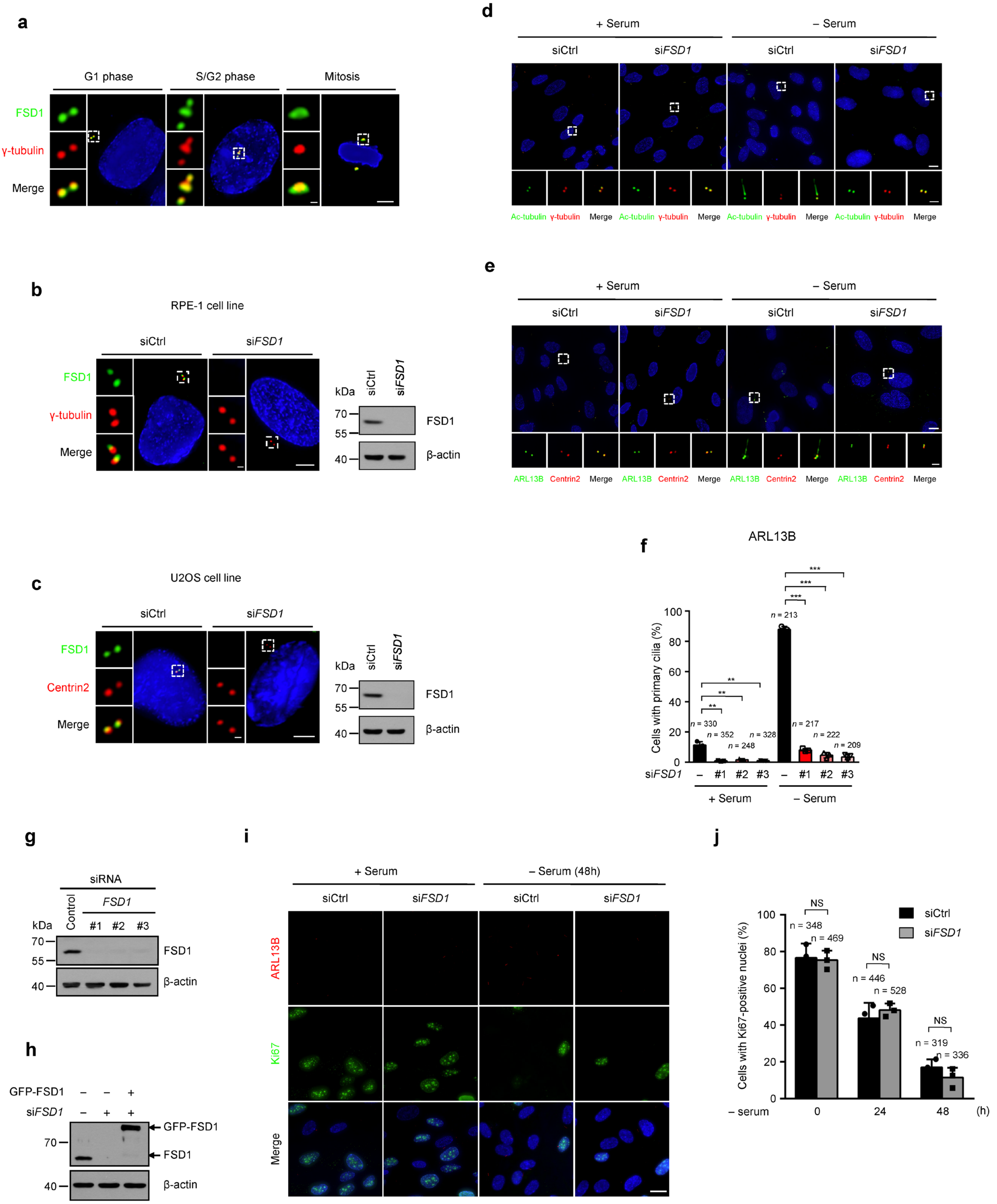

Supplementary Figure 1. FSD1 Localized at Centrosome Is Required for Ciliogenesis.
(a) FSD1 is present at centrioles throughout the cell cycle. RPE-1 cells in different cell-cycle stages were processed for staining with the indicated antibodies. Magnified centrioles are shown in the insets. Scale bars, 5 μm (main image) and 500 nm (magnified region). **(b, c)** RPE-1 or U2OS cells

were transfected with control or FSD1 siRNA for 72 h, then processed for immunofluorescence and Western blot analyses with indicated antibodies. DNA was stained with Hoechst (blue). β-actin was used as a loading control. Magnified centrioles are shown in the insets. Scale bars, 5 μm (main image) and 500 nm (magnified region). **(d)** Representative images of cycling (+Serum) or quiescent (-Serum) RPE-1 cells transfected with indicated siRNA in Fig. 1b. Acetylated α-tubulin (Ac-tubulin) is ciliary marker. Scale bars, 5 μm (main image) and 2 μm (magnified region). **(e, f)** Effects of FSD1 depletion on cilia formation in cycling (+Serum) or quiescent (-Serum) RPE-1 cells. ADP ribosylation factor like GTPase 13B (ARL13B) is ciliary marker. Scale bars, 5 μm (main image) and 2 μm (magnified region). **(g)** Immunoblots of RPE-1 cell lysates in Fig. 1b with the indicated antibodies. β-actin was used as a loading control. **(h)** Immunoblots of RPE-1 cell lysates in Fig. 1c with the indicated antibodies. β-actin was used as a loading control. **(i)** Cycling (+Serum) or quiescent (-Serum) RPE-1 cells were transfected with control or FSD1 siRNAs for 48 h and stained with indicated antibodies as shown. Scale bar, 20 μm. **(j)** The percentage of Ki67-positive RPE-1 cells treated with control or FSD1 siRNA at indicated time points was determined based on (i). Data are presented as mean ± s.d. of at least three independent experiments. *n*, number of cells.

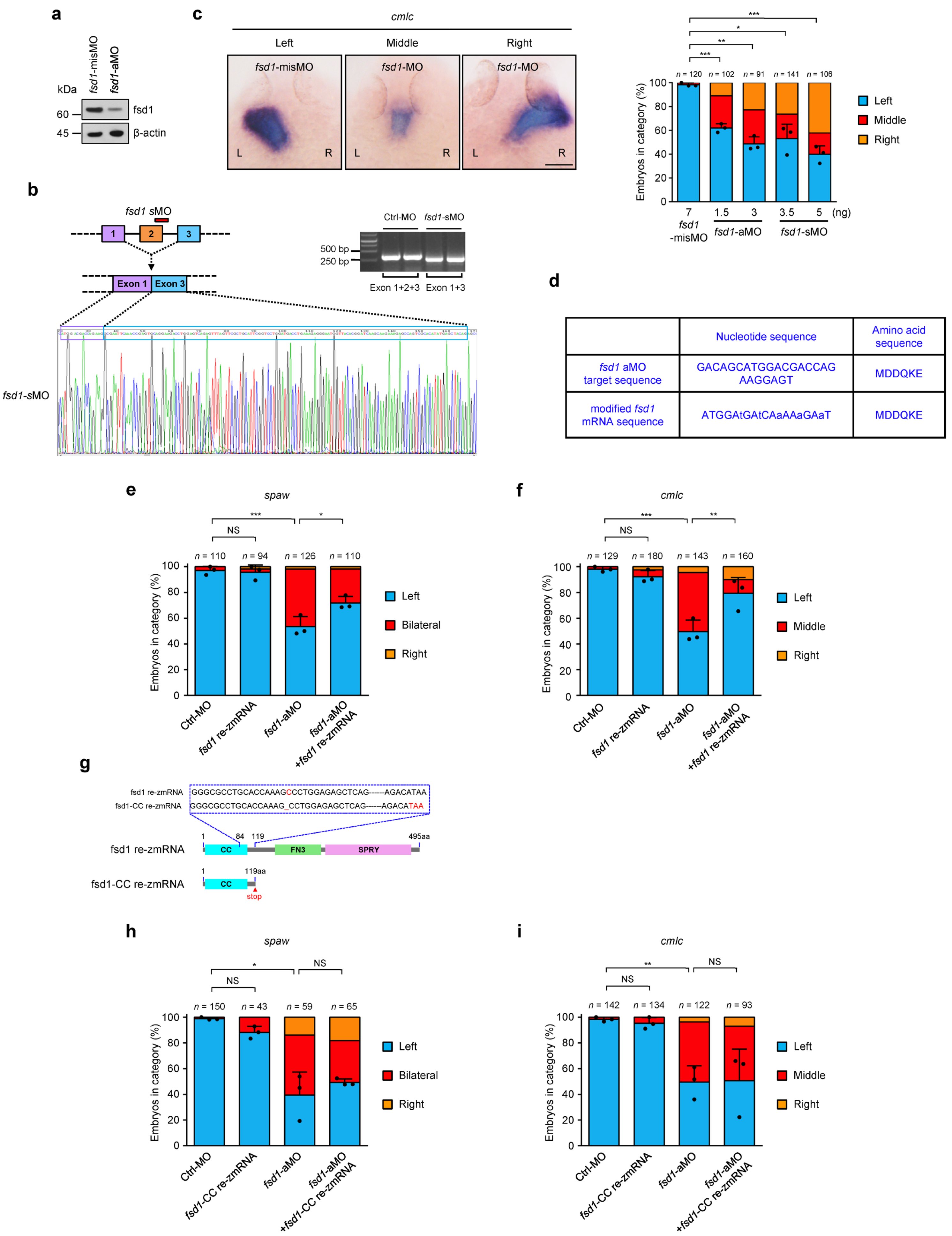

	Nucleotide sequence	Amino acid sequence
fsd1 aMO target sequence	GACAGCATGGACGACCAG AAGGAGT	MDDQKE
modified *fsd1* mRNA sequence	ATGGAtGAtCAaAAaGAaT	MDDQKE

Supplementary Figure 2. Knockdown of fsd1 Affects Cilia Formation and Laterality Development in Zebrafish.

(a) *Fsd1* atg-morpholino efficiency was validated by western blot. **(b)** *Fsd1* splice-morpholino efficiency was validated by RT-PCR and sequencing. The PCR product was 292 bp in control embryos and 194 bp in *fsd1* sMO injected embryos. The sequencing result of the PCR product below showed deletion of exon 2 (98 bp) in *fsd1* sMO injected embryos. Purple, orange and blue colors mark exon 1, 2 and 3, respectively. The red box indicates the *fsd1* sMO targeted sequence. **(c)** *Fsd1* MOs caused left-right asymmetry defects. The *cmlc* probe was used to label the heart tube in the whole-mount in situ hybridization at 26 hpf. Scale bar, 100 μm. *n*, number of fishes. **(d)** The target sequence of *fsd1* aMO and sequence of aMO-resistant form of zebrafish fsd1 mRNA (*fsd1* re-zmRNA). **(e, f)** Zebrafish *fsd1* mRNA partially rescued the LR asymmetry defects induced by *fsd1* aMO marked by *spaw* or *cmlc*. **(g)** Schematic diagram illustrates the difference between *fsd1* re-zmRNA and *fsd1*-CC re-zmRNA. **(h, i)** Zebrafish *fsd1*-CC re-zmRNA could not rescue the LR asymmetry defects induced by *fsd1* aMO marked by *spaw* or *cmlc*. Data are presented as mean ± s.d. of at least three independent experiments. NS, not significant, *P < 0.05, **P < 0.01, ***P < 0.001. *n*, number of fishes.

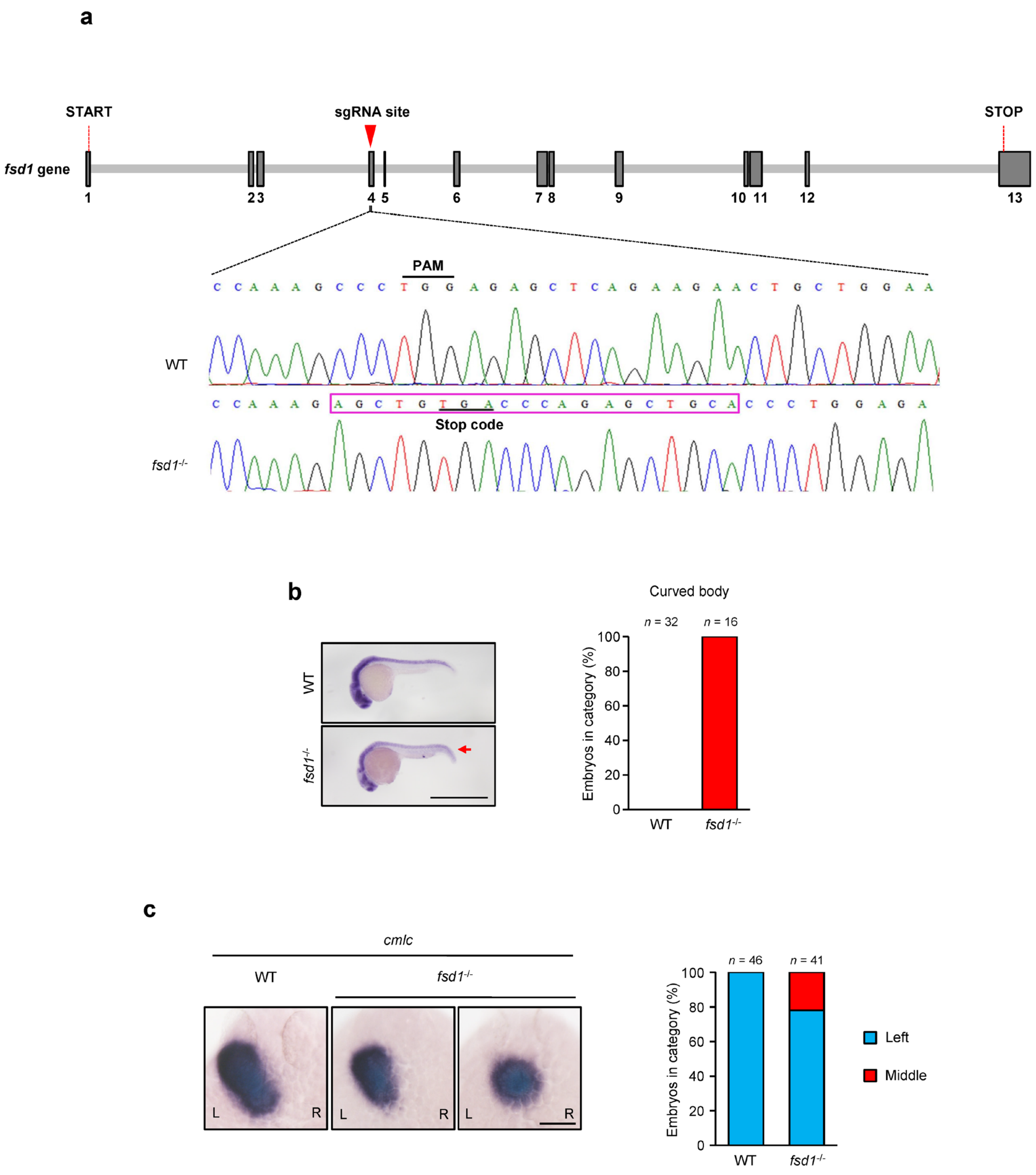

Supplementary Figure 3. Knock out of fsd1 affects laterality development in zebrafish.
(a) *Fsd1* knock out efficiency was validated by sequencing. **(b)** *Fsd1* knock out displayed curved body at 72 hpf. The arrow marked curved body. Scale bars, 1 mm. **(c)** *Fsd1* knock out caused left-right asymmetry defects. The *cmlc* probe was used to label the heart tube in the whole-mount in situ hybridization at 26 hpf. Scale bar, 100 μm. *n*, number of fishes.

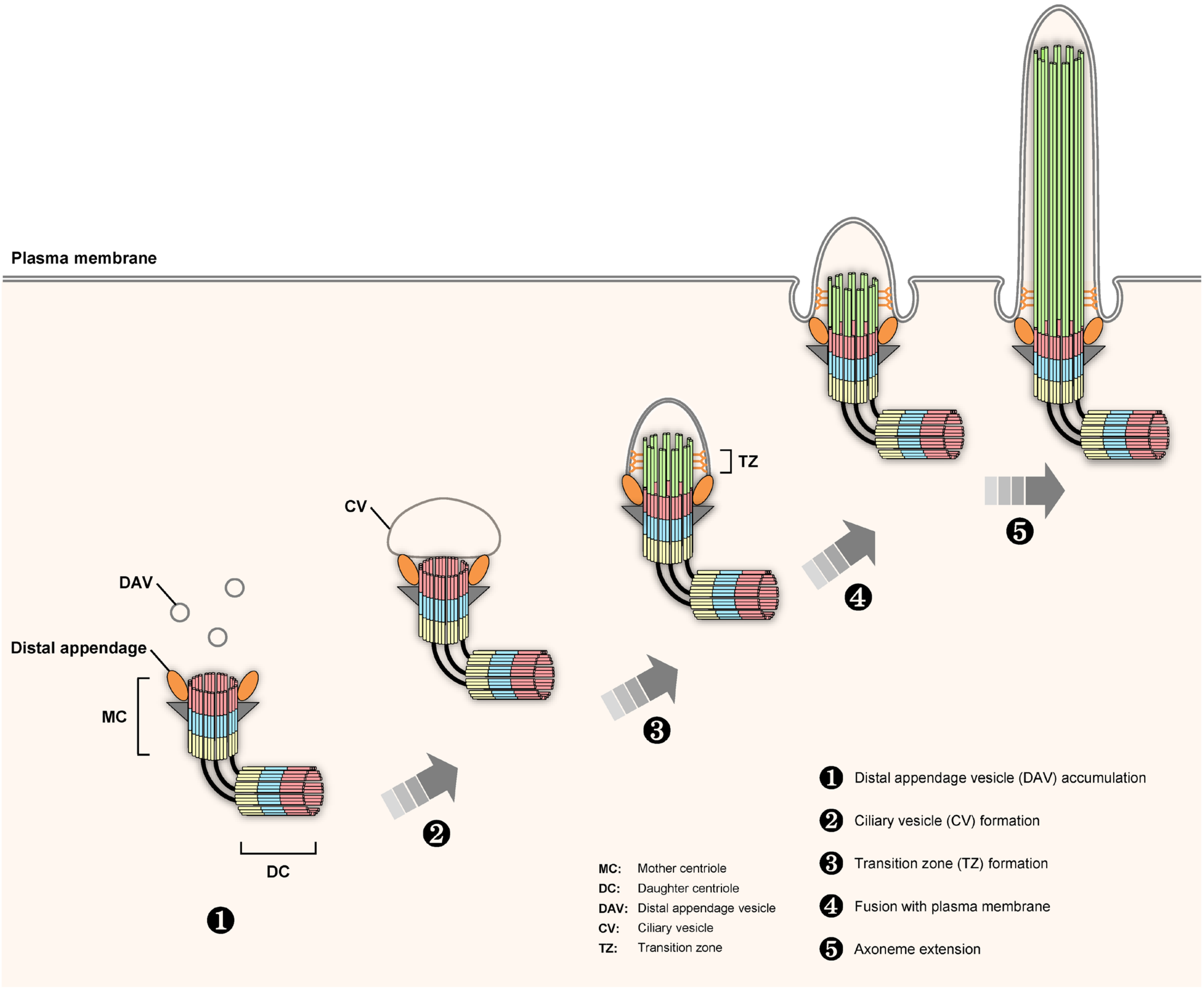

Supplementary Figure 4. The Multiple Steps of Ciliogenesis.
During ciliogenesis, small cytoplasmic vesicles termed distal appendage vesicle (DAV) accumulate, fuse and then produce a ciliary vesicle (CV) on the distal tip of mother centrioles (basal body). This process then facilitates initial microtubules to crosslink with the CV membrane to subsequently form a specialized compartment described as the transition zone (TZ). After transition zone formation, the cilium's microtubules then grow into this vesicular cap which eventually fuses with the cellular plasma membrane, thereby exposing the cilium to the extracellular environment.

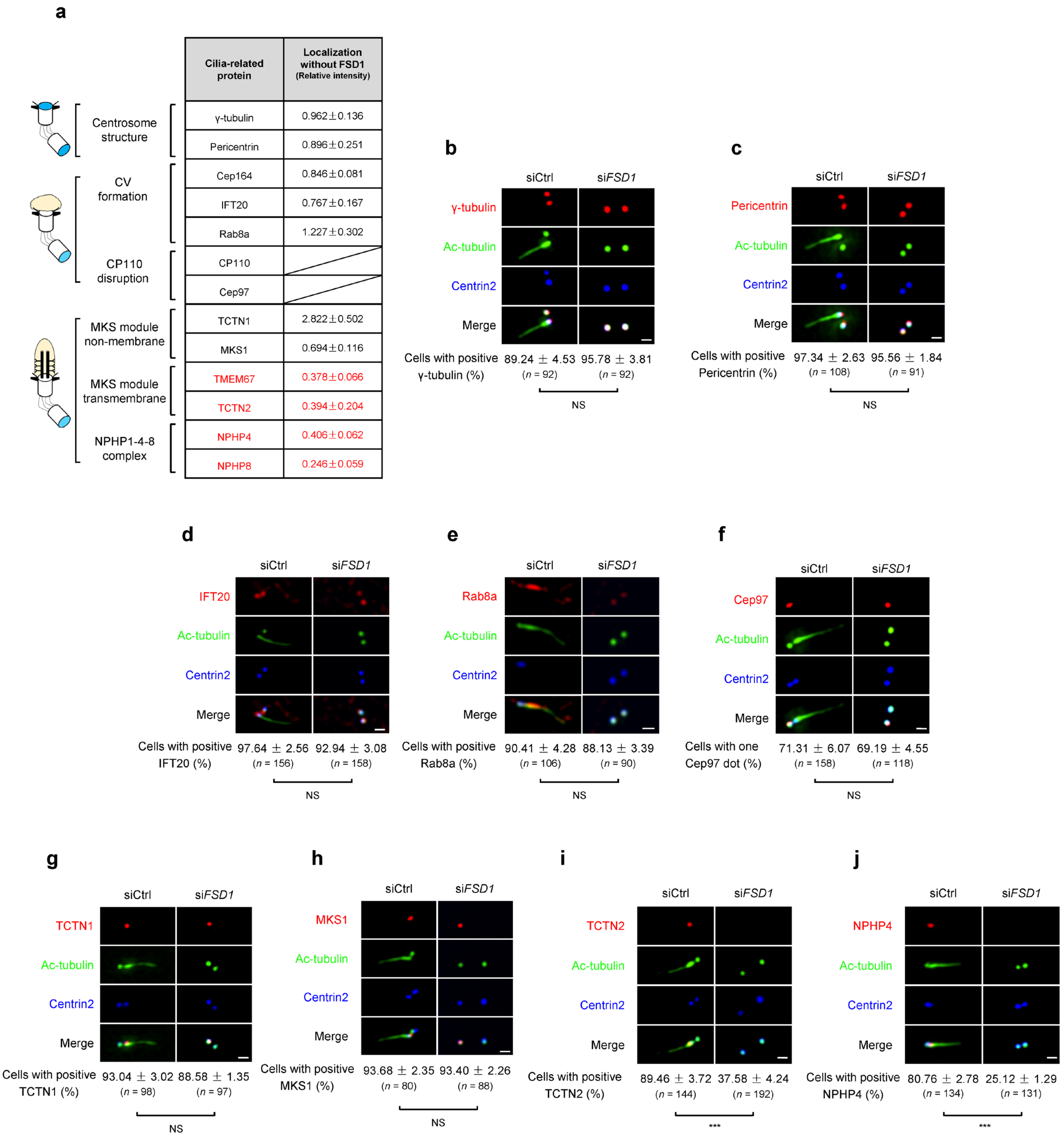

Supplementary Figure 5. FSD1 Is Required for TZ Component Recruitment, but Could Not Affect the Centrosome Structure and Ciliary Vesicle Formation.

(a) The quantification of the relative intensity of the tested proteins at centrioles without FSD1. CV, ciliary vesicle. **(b-j)** RPE-1 cells were treated with control or FSD1 siRNAs followed by serum starvation for 48 h, then stained with indicated antibodies. (b, c) FSD1 is dispensable for the γ-tubulin and pericentrin localization at centrioles. (d, e) FSD1 depletion could not affect IFT20 and Rab8a localization at centrioles. (f) FSD1 depletion could not affect the disruption of Cep97 at mother centriole. (g, h) The localization of TCTN1 and MKS1 at centrioles were not affected after

FSD1 depletion. (i, j) The loss of FSD1 could affect the localization of transition zone component TCTN2 and NPHP4. Scale bars, 1 μm. Data are presented as mean ± s.d. of three independent experiments. NS, not significant, ***P < 0.001. n, number of cells.

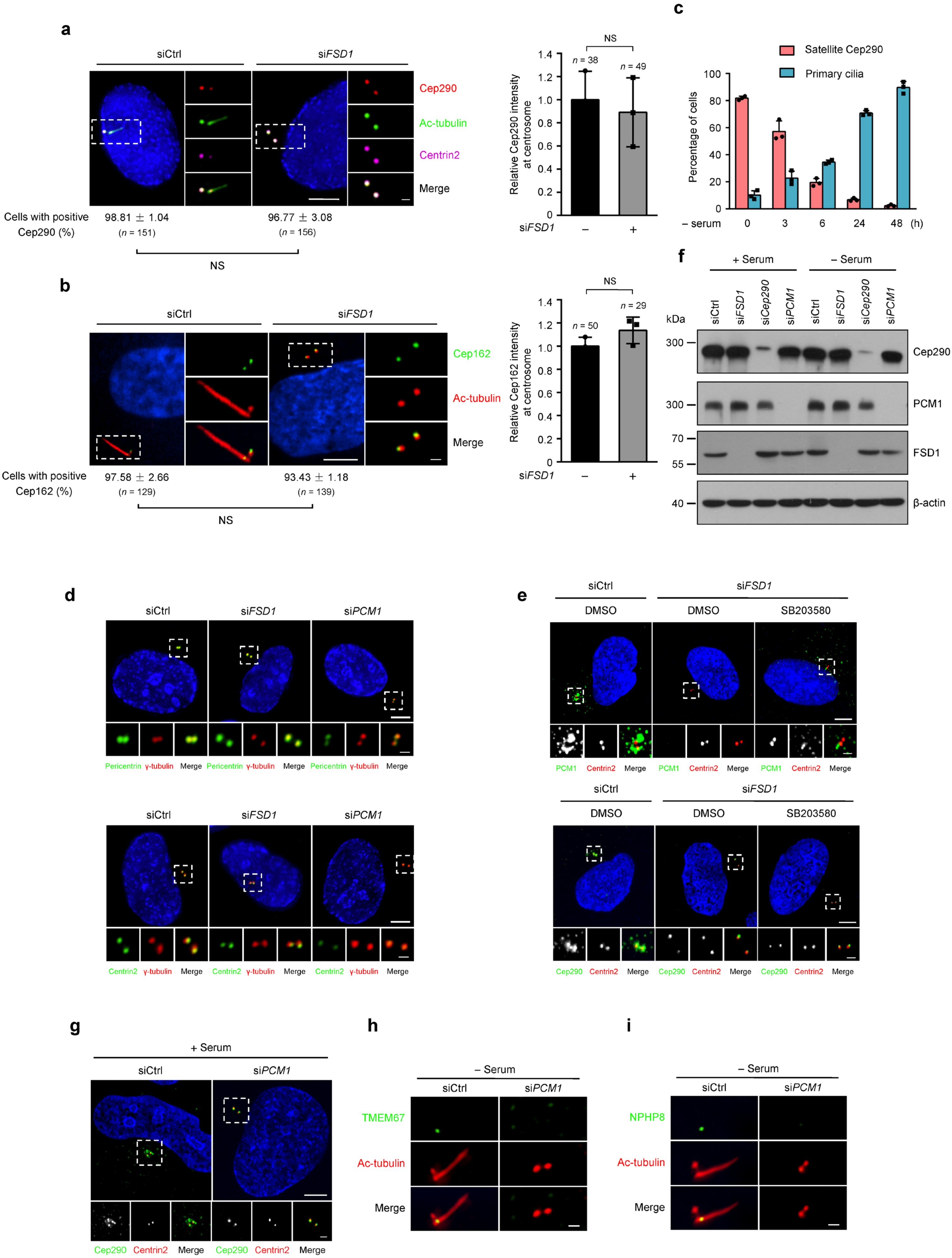

a
siCtrl
siFSD1
Cep290
Ac-tubulin
Centrin2
Merge
Cells with positive Cep290 (%)
98.81 ± 1.04
(n = 151)
96.77 ± 3.08
(n = 156)
NS
Relative Cep290 intensity at centrosome
n = 38
n = 49
siFSD1
c
Satellite Cep290
Primary cilia
Percentage of cells
– serum
(h)
b
Cep162
Ac-tubulin
Merge
Cells with positive Cep162 (%)
97.58 ± 2.66
(n = 129)
93.43 ± 1.18
(n = 139)
Relative Cep162 intensity at centrosome
n = 50
n = 29
f
+ Serum
– Serum
kDa
siCtrl
siFSD1
siCep290
siPCM1
Cep290
PCM1
FSD1
β-actin
d
siPCM1
Pericentrin
γ-tubulin
Centrin2
e
DMSO
SB203580
PCM1
Cep290
g
h
TMEM67
i
NPHP8

Supplementary Figure 6. PCM1 Is Required for Transition Zone Assembly.
(a, b) RPE-1 cells were treated with control or FSD1 siRNAs followed by serum starvation for 48 h, then stained with indicated antibodies. Scale bars, 5 μm (main image) and 1 μm (magnified region). (a) FSD1 depletion could not affect the Cep290 localization at centrioles. (b) FSD1 depletion could not affect the Cep162 localization at centrioles. **(c)** The percentage of RPE-1 cells with primary cilia or accumulation of Cep290 granules around centrosomes were determined in Fig. 4a. **(d)** RPE-1 cells transfected with control, FSD1 or PCM1 siRNAs were stained with indicated antibodies. Scale bar, 5 μm. **(e)** RPE-1 cells transfected with control or FSD1 siRNAs were incubated with 10 uM SB203580 (p38 inhibitor) overnight. Next, cells were fixed and stained with indicated antibodies. Scale bar, 5 μm. **(f)** Western blot analyses of protein extracts from RPE-1 cells transfected with indicated siRNA. The protein levels of Cep290, PCM1 and FSD1 were detected with indicated antibodies. β-actin was used as a loading control. **(g)** Representative images of RPE-1 cells transfected with indicated siRNA in Fig. 4d. Scale bar, 5 μm. **(h)** Representative images of RPE-1 cells transfected with indicated siRNA in Fig. 4e. Scale bar, 1 μm. **(i)** Representative images of RPE-1 cells transfected with indicated siRNA in Fig. 4f. Scale bar, 1 μm. Data are presented as mean ± s.d. of three independent experiments. NS, not significant. *n*, number of cells.

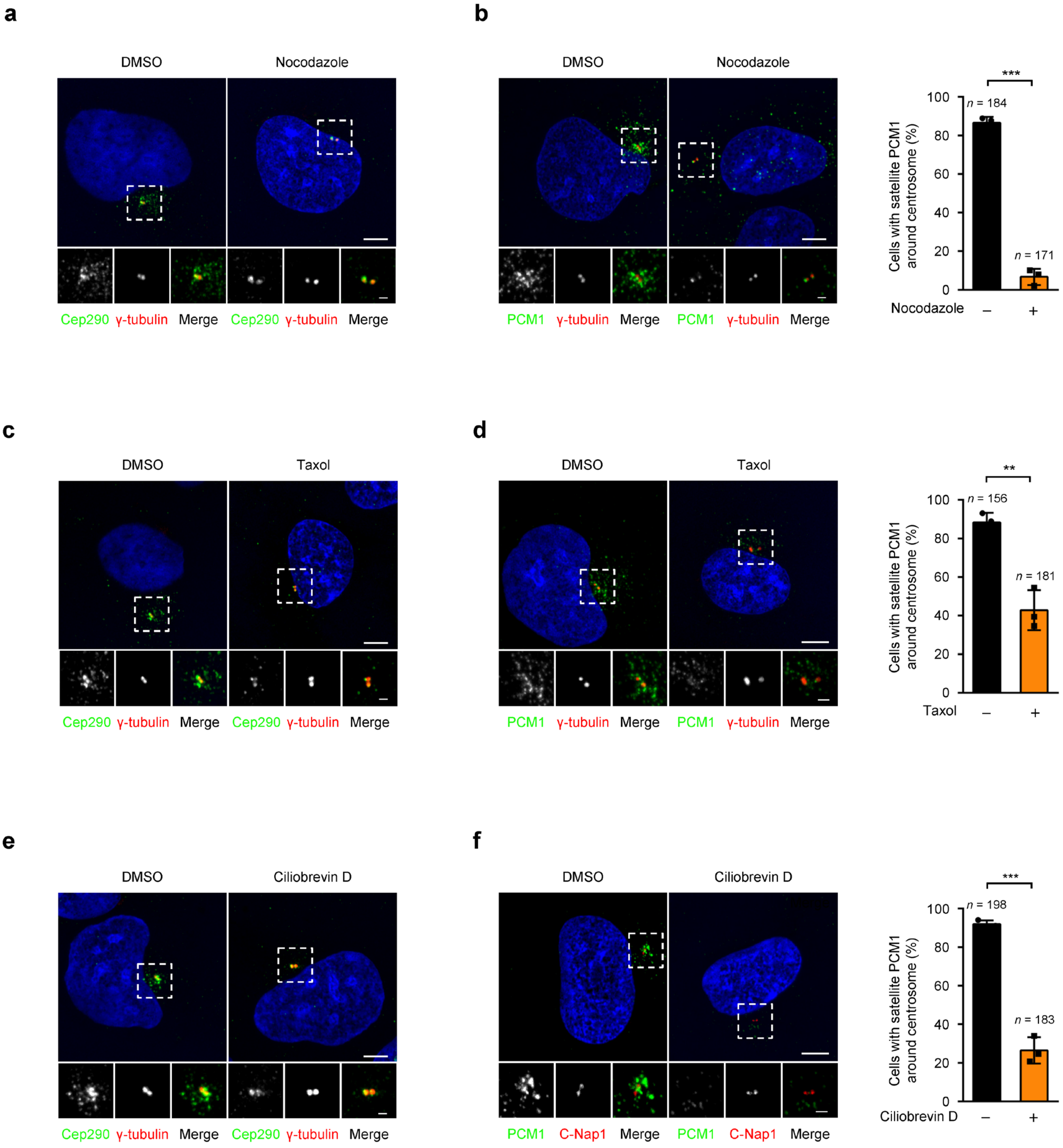

Supplementary Figure 7. Microtubule Dynamics are Required for CS Cep290 and PCM1 Localization.

(a) Representative images of RPE-1 cells treated with DMSO or 20 μM nocodazole in Fig. 5a. **(b)** RPE-1 cells treated with DMSO or 20 μM nocodazole for 30 min were stained with indicated antibodies. Percentage of cells with CS PCM1 was shown on the right panel. **(c)** Representative images of RPE-1 cells treated with DMSO or 1 μM taxol in Fig. 5b. **(d)** RPE-1 cells treated with DMSO or 1 μM taxol for 30 min were stained with indicated antibodies. Percentage of cells with CS PCM1 was shown on the right panel. **(e)** Representative images of RPE-1 cells treated with DMSO or 10 μM ciliobrevin D in Fig. 5c. **(f)** RPE-1 cells treated with DMSO or 20 μM ciliobrevin D for 30 min were stained with indicated antibodies. Percentage of cells with CS PCM1 was shown on the right panel. Magnified centrioles are shown in the insets. Scale bars, 5 μm (main image) and 1 μm (magnified region). Data are presented as mean ± s.d. of three independent experiments. $^{**}P < 0.01$, $^{***}P < 0.001$. *n*, number of cells.

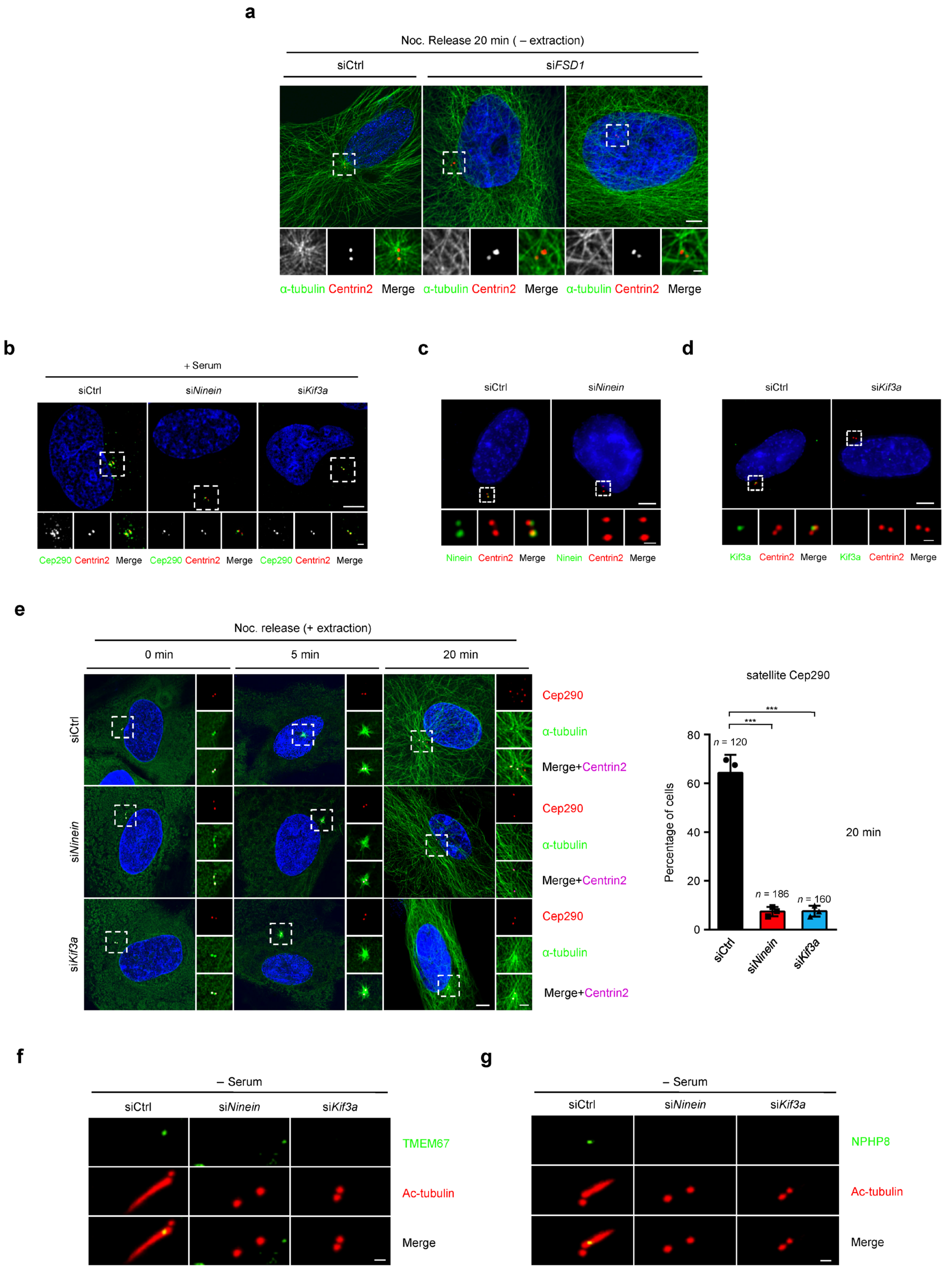
a
Noc. Release 20 min (– extraction)
siCtrl
siFSD1
α-tubulin Centrin2 Merge α-tubulin Centrin2 Merge α-tubulin Centrin2 Merge
b
+ Serum
siCtrl
siNinein
siKif3a
Cep290 Centrin2 Merge Cep290 Centrin2 Merge Cep290 Centrin2 Merge
c
siCtrl
siNinein
Ninein Centrin2 Merge Ninein Centrin2 Merge
d
siCtrl
siKif3a
Kif3a Centrin2 Merge Kif3a Centrin2 Merge
e
Noc. release (+ extraction)
0 min
5 min
20 min
siCtrl
siNinein
siKif3a
Cep290
α-tubulin
Merge+Centrin2
satellite Cep290
Percentage of cells
n = 120
n = 186
n = 160
***
***
20 min
siCtrl
siNinein
siKif3a
f
– Serum
siCtrl
siNinein
siKif3a
TMEM67
Ac-tubulin
Merge
g
– Serum
siCtrl
siNinein
siKif3a
NPHP8
Ac-tubulin
Merge

Supplementary Figure 8. Depletion of Ninein or Kif3a Caused TZ Assembly Defects.

(a) Other representative images of RPE-1 cells transfected with indicated siRNA after nocodazole release 20 min in Fig. 5d. Scale bars, 5 μm (main image) and 1 μm (magnified region). **(b)** Representative images of RPE-1 cells treated with indicated siRNA in Fig. 5f. Scale bars, 5 μm (main image) and 1 μm (magnified region) **(c, d)** The knockdown efficiency of Ninein and Kif3a in Fig. 5g. Scale bars, 5 μm (main image) and 1 μm (magnified region). **(e)** RPE-1 cells were transfected with indicated siRNA and subjected to a microtubule regrowth assay at the indicated time points, fixed and permeabilized with cold methanol and stained with antibodies to Cep290 (red), α-tubulin (green) and centrin2 (purple). Scale bars, 5 μm (main image) and 2 μm (magnified region). Percentage of cells with CS Cep290 after nocodazole release 20 min was quantified on the right of cells. **(f)** Representative images of RPE-1 cells treated with indicated siRNA in Fig. 5g. Scale bar, 1 μm. **(g)** Representative images of RPE-1 cells treated with indicated siRNA in Fig. 5h. Scale bar, 1 μm.

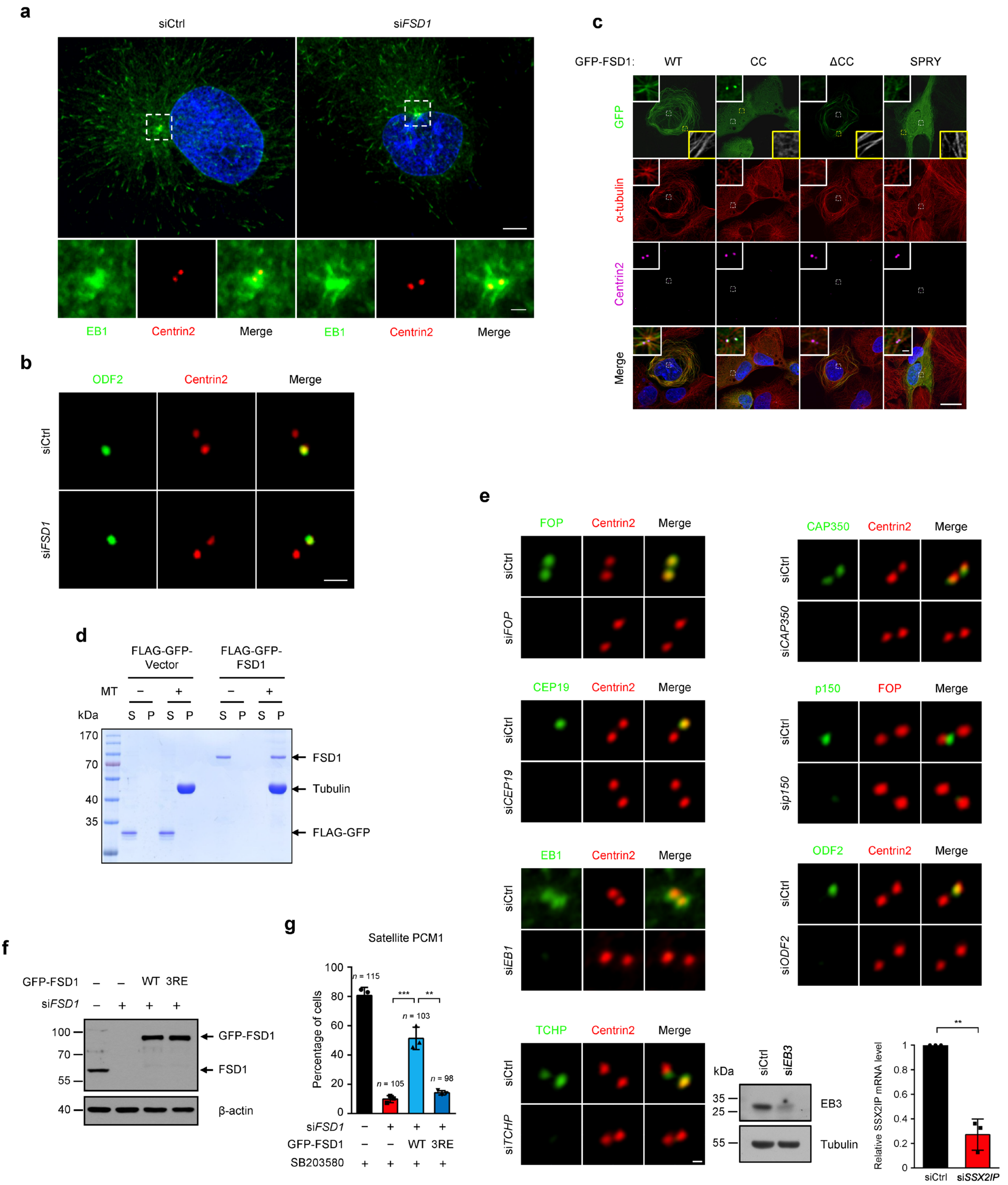

Supplementary Figure 9. FSD1 does not Affect Other Anchorage Protein Localization.
(a) RPE-1 cells transfected with control or FSD1 siRNAs were stained with indicated antibodies. Magnified centrioles are shown in the insets. Scale bars, 5 µm (main image) and 1 µm (magnified region). **(b)** RPE-1 cells transfected with control or FSD1 siRNAs were stained with indicated antibodies. Scale bar, 1 µm. **(c)** The distinct domains of FSD1 mediate its centrosome localization and MT association. RPE-1 cells expressing GFP-tagged fragments of FSD1 were stained with the

antibodies indicated. Magnified centrioles are shown in the insets. Scale bars, 20 μm (main image) and 1 μm (magnified region). **(d)** Purified FLAG-GFP-FSD1, but not FLAG-GFP-Vector, was co-pelleted with Taxol-stabilized microtubules. **(e)** The knockdown efficiency of indicated siRNA in Fig. 7a was validated by immunofluorescence, western blot or qPCR (relative expression compared to β-actin). Scale bar, 500 nm. **(f)** Western blot analyses of protein extracts from RPE-1 cells transfected with indicated siRNA and plasmid. The protein levels were detected with anti-FSD1 antibody. β-actin was used as a loading control. **(g)** Expression of GFP-FSD1 WT, but not 3RE mutant, rescued PCM1 localization at centriolar satellites in cycling cells with the addition of 50 uM SB203580 (p38 inhibitor).

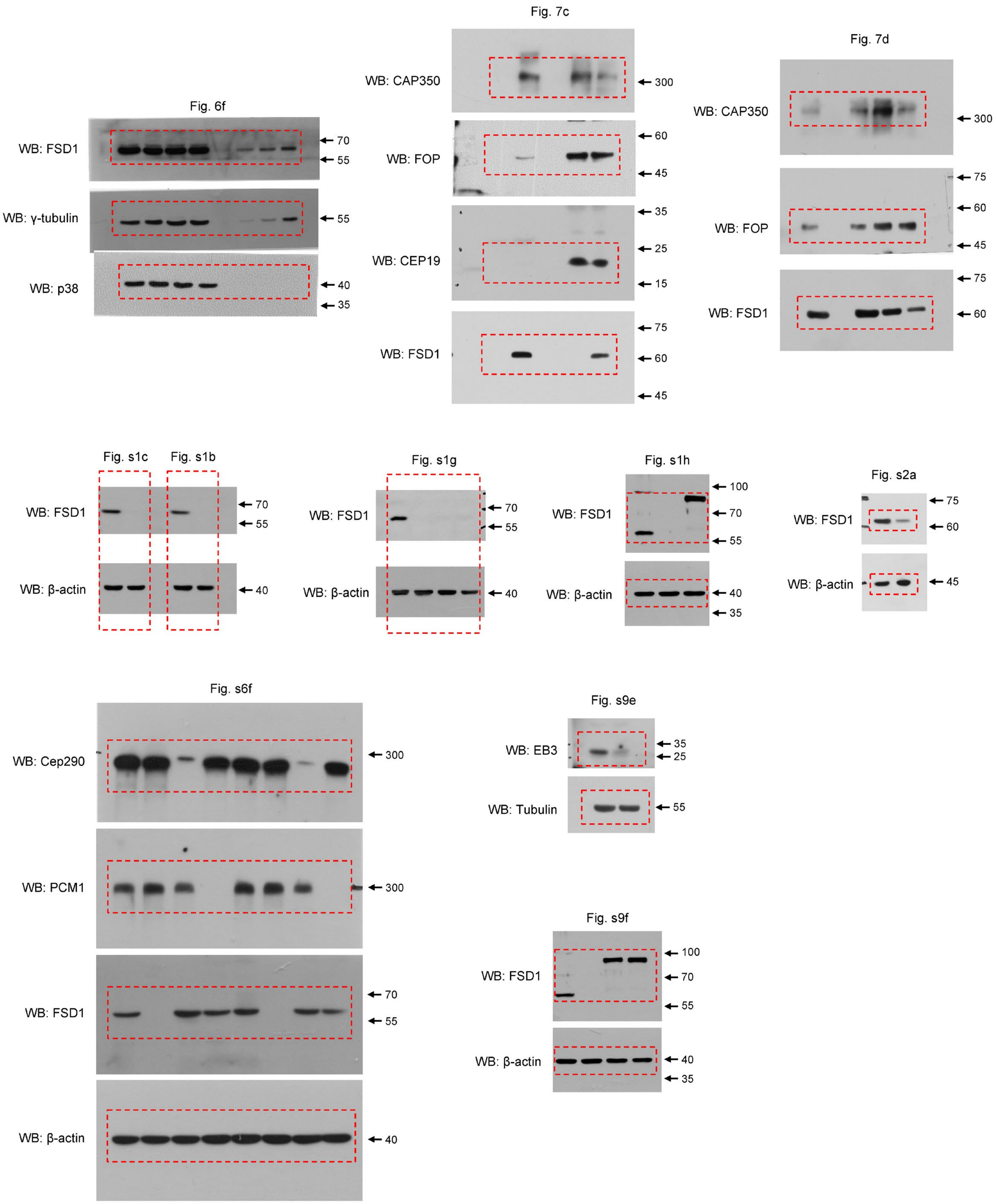

Supplementary Figure 10. **Unprocessed images of blots.**

Unprocessed images of scanned immunoblots shown in Figure 6, 7 and Supplementary Figure 1, 2, 6, 9 are provided.
